# Predisposition, Insult/Infection, Response and Organ Dysfunction (PIRO): A Pilot Clinical Staging System for Hospital Mortality in Patients with Infection

**DOI:** 10.1371/journal.pone.0070806

**Published:** 2013-07-24

**Authors:** Teresa Cardoso, Armando Teixeira-Pinto, Pedro Pereira Rodrigues, Irene Aragão, Altamiro Costa-Pereira, António E. Sarmento

**Affiliations:** 1 Intensive Care Unit, Unidade de Cuidados Intensivos Polivalente, Hospital de Santo António, University of Porto, Porto, Portugal; 2 Department of Health Information and Decision Sciences, Center for Research in Health Technologies and Information Systems (CINTESIS), Faculty of Medicine, University of Porto, Porto, Portugal; and Sydney School of Public Health, Faculty of Medicine, University of Sydney, Sydney, Australia; 3 Department of Health Information and Decision Sciences, Center for Research in Health Technologies and Information Systems (CINTESIS), Faculty of Medicine, University of Porto, Porto, Portugal; 4 Unidade de Cuidados Intensivos Polivalente, Hospital de Santo António, University of Porto, Porto, Portugal; 5 Department of Health Information and Decision Sciences, Center for Research in Health Technologies and Information Systems (CINTESIS), Faculty of Medicine, University of Porto, Porto, Portugal; 6 Department of Infectious Diseases, Hospital de São João, University of Porto, Porto, Portugal; University of Leicester, United Kingdom

## Abstract

**Purpose:**

To develop a clinical staging system based on the PIRO concept (Predisposition, Infection, Response and Organ dysfunction) for hospitalized patients with infection.

**Methods:**

One year prospective cohort study of all hospitalized patients with infection (n = 1035), admitted into a large tertiary care, university hospital. Variables associated with hospital mortality were selected using logistic regressions. Based on the regression coefficients, a score for each PIRO component was developed and a classification tree was used to stratify patients into four stages of increased risk of hospital mortality. The final clinical staging system was then validated using an independent cohort (n = 186).

**Results:**

Factors significantly associated with hospital mortality were • for Predisposition: age, sex, previous antibiotic therapy, chronic hepatic disease, chronic hematologic disease, cancer, atherosclerosis and a Karnofsky index<70; • for Insult/Infection: type of infection • for Response: abnormal temperature, tachypnea, hyperglycemia and severity of infection and • for Organ dysfunction: hypotension and SOFA score≥1. The area under the ROC curve (CI_95%_) for the combined PIRO model as a predictor for mortality was 0.85 (0.82–0.88). Based on the scores for each of the PIRO components and on the cut-offs estimated from the classification tree, patients were stratified into four stages of increased mortality rates: stage I: ≤5%, stage II: 6–20%, stage III: 21–50% and stage IV: >50%. Finally, this new clinical staging system was studied in a validation cohort, which provided similar results (0%, 9%, 31% and 67%, in each stage, respectively).

**Conclusions:**

Based on the PIRO concept, a new clinical staging system was developed for hospitalized patients with infection, allowing stratification into four stages of increased mortality, using the different scores obtained in Predisposition, Response, Infection and Organ dysfunction. The proposed system will likely help to define inclusion criteria in clinical trials as well as tailoring individual management plans for patients with infection.

## Introduction

According to the 2012 World Health Organization report, infections are among the top three leading causes of death worldwide [1]. Developing new therapies for sepsis has been particularly challenging and the successive failures have been attributed to the inclusion of a very heterogeneous group of patients.

In 2001, the American College of Chest Physicians and the Society of Critical Care Medicine convened a consensus panel, where John Marshall *et al* [2]. suggested an approach of sepsis similar to the TNM (tumor, nodes and metastases) staging system [3], used for cancer patients both as a prognostic tool and for individualizing therapy. This led to the PIRO concept, which attempts to characterize sepsis across four components: P for ‘Predisposition (P), I for ‘Insult/Infection (I), R for ‘Response (R) and O for ‘Organ dysfunction (O) [2], [4].

This challenging concept took some time before being adopted by the scientific community and was only recently tested in the clinical field. Different approaches have been published, a model development [5], [6] and a scoring system [7]–[9] solely for patients in the intensive care unit (ICU) setting; a score for patients with suspected infection admitted from the Emergency department [10] and finally a study that developed a staging system [11] but solely for patients with severe sepsis and not considering all the originally proposed variables.

The need for a clinical staging system applicable to all hospitalized patients with confirmed infection remained. This would help stratifying patients at risk, assess criteria for specific therapies, predict outcomes and assist in rational enrolment into clinical studies.

The objective of this study was to develop a clinical staging system based on the PIRO concept through a prospective cohort study in a diverse population of patients with infection on hospital admission or throughout their hospital stay. The derived clinical staging system is validated in an independent cohort.

## Patients and Methods

A prospective cohort study was conducted in a 600-bed tertiary care university hospital over a 1-year period (1^st^ June 2008 to 31^th^ May 2009). All adult (age≥18 years) infected patients consecutively admitted to the medical, surgical, nephrology or hematology wards of the hospital, or to ICU were included in the first 24 hours of the diagnosis of infection according to the CDC criteria [12]. The inclusion criteria did not include microbiological data in order to obviate delay in study inclusion or retrospective data collection for diagnosis confirmation.

The data collected included all variables defined in the extended sepsis criteria [2] which were grouped according to each PIRO component as follows.

**1.** **Predisposing factors (‘P’)** analyzed included: age, sex, season of admission, previous antibiotic therapy (any antibiotic administration with therapeutic intention in the previous month), hospitalization in the previous year, previous instrumentation, Karnofsky index [13] (a value lower than 70 means inability to carry out normal activity or do active work) and premorbid conditions. Chronic morbidities recorded were: immunosuppression (administration of chemotherapy, radiation therapy during 12 months prior to hospital admission or the equivalent of 0.2 mg/Kg/day prednisolone for at least 3 months or 1 mg/Kg/day for a week during 3 months prior to hospital admission or human immunodeficiency virus infection), chronic hepatic disease [14], chronic heart failure [14], chronic respiratory disease [14], hematologic disease [15], cancer [15], chronic renal failure (if there was need for chronic renal support or a history of chronic renal insufficiency with a serum creatinine level over 2 mg/dl), diabetes *mellitus* (if insulin therapy or oral anti-diabetic drugs were required before the infection) and/or atherosclerosis (if there was a previous history of transient ischemic attack, stroke, angina, myocardial infarction or peripheral arterial disease).

**2. Insult/Infection** **(‘I’)** was characterized by: type of infection, categorized as either community-acquired (CAI), if the infection was detected within 48 hours of hospital admission in patients who did not fulfill the criteria for a healthcare-associated infection; healthcare associated (HCAI - using the same criteria that Deborah Friedman used for healthcare associated bloodstream infections regardless of the involved focus of infection) [16] or hospital-acquired (HAI) [12]; focus of infection (categorized as respiratory [12], urinary [12], intra-abdominal [12], or others); microbiology documentation of infection; presence of bacteremia (primary or secondary) [17] and pathogen identification, classified by category (Gram negative, Gram positive, fungus or poly-microbial).

**3. Host Response variables (‘R’)** included: abnormal temperature (fever or hypothermia), tachypnea, tachycardia, abnormal white blood cells count (leukocytosis, leucopenia), altered consciousness, hyperglycemia in the absence of diabetes, peripheral edema, high serum C-reactive protein and severity of infection as defined in the 2001 SCCM/ESICM/ACCP/ATS/SIS International Sepsis Definitions Conference (infection, sepsis, severe sepsis or septic shock at presentation [2]).

**4. Organ dysfunction (‘O’)** was assessed by the following variables: hypoxemia, hypotension, high serum lactate, renal dysfunction, high bilirubin, low platelet count, ileus, coagulopathy and total SOFA score [18].

A second cohort was established to validate the proposed clinical staging system. Data for the validation cohort were retrospectively collected and included all patients admitted to the same wards between 1^st^ December 2011 and 31^st^ January 2012 as the derivation cohort, using the same inclusion criteria.

The **primary outcome of interest** was on in-hospital mortality. All patients had complete follow-up until hospital discharge in both cohorts.

This study was approved by the institutional review board (which includes the Ethics for Health Committee) of Hospital de Santo António, Oporto Hospital Centre, Portugal, and informed consent from the participants was waived due to its purely observational nature.

### Statistical Analysis

Continuous variables were described as means and standard deviations (SD) or as medians and inter-quartile ranges (IQR) if they showed a skewed distribution. Categorical variables were described with absolute frequencies and percentages. Student *T*-tests or Mann-Whitney tests were used to compare continuous values between survivors and non-survivors. For categorical variables, these comparisons were performed using Pearson χ^2^ test.

To build the prediction models for P, I, R and O, variables with marginal association with mortality in the univariate analysis (*p* value <0.2) were screened for the multivariate analysis. Four separate logistic regression models - one for each component “P”, “I”, “R” and “O” – were built using stepwise selection on the variables screened in the previous phase. Once the models were fitted, four scores for each patient were computed, representing the probability of death predicted by each model. The four scores were combined into a logistic regression referred to as “combined PIRO”.

The results of the multivariable models are expressed as odds ratio (OR) with 95% confidence interval (CI_95%_) and p-values. The accuracy of the models was assessed by the area under receiver operating characteristics curve (AU-ROC) and calibration was tested using the Hosmer-Lemeshow goodness-of-fit test.

In order to simplify the computation of the scores for each component, the regression coefficients were multiplied by two and rounded to the nearest integer. This simplified scoring system was then used to compare the scores obtained directly from the derivation models with the non-rounded coefficients. The AU-ROCs of the simplified version were identical to the derivation ones.

After obtaining the new scores for each PIRO component, a classification tree was used to define cut-offs for component score and identify profiles of risk of death across the four PIRO components. Each node split decision in the tree was chosen from the possible cut-offs for all components, maximizing the within-node homogeneity according to Gini's coefficient [19] impurity measure, which is known to be closely related to both, the AU-ROC and the Mann-Whitney-U test [20].

A cross table with all possible profiles resulting from the combination of Predisposition, Infection, Response and Organ dysfunction against hospital outcome was built and analyzed to yield the final algorithm. In order to simplify the presentation of results, the profiles were further clustered into four clinical stages according to the risk of death.

The significance level for all tests was defined as p<0.05. Data were analyzed using SPSS, version 18 for Windows (Chicago, IL).

## Results

During the study period, a total of 3733 patients were assessed and 1035 (28%) met the inclusion criteria for having infection according to the CDC definitions of infection and hence were included in the study. Mean (SD) age was 65 (20) years and mean SAPS II was 29 (13), overall hospital mortality rate was 13% (table 1). The median hospital length of stay in the derivation cohort was 11 (7–22) days. A microbiological confirmation of infection was available in 68% of patients (56% for CAI, 73% for HCAI and 83% for HAI).The validation cohort included 186 patients that were significantly older, with a mean age of 69 (17) years and a mean SAPS II of 35 (15) (table 1).

**Table 1:** Demographic and clinical characteristics of patients in the derivation and validation cohorts.

Variables	Derivation cohort (n = 1035)	Validation cohort (n = 186)	p- value
Age in years, mean (SD)	65 (20)	69 (17)	0.002*
Male sex, n (%)	506 (49)	109 (59)	0.015#
ICU patients	149 (14)	40 (22)	0.016#
SAPS II, mean (SD)	29 (13)	35 (15)	<0.003*
Total SOFA, median (IQR)	1 (0–3)	2 (1–4)	<0.001&
Hospital mortality, n (%)	138 (13)	34 (18)	0.085#

SD – standard deviation, IQR – interquartile range. SOFA – Sepsis-related Organ Failure Assessment

* Independent samples *t-*test,

# Pearson Cui-square Test;

& Independent samples median test.

In table 2, a detailed description of patients’ characteristics and their association with hospital mortality, according to the four components of PIRO, is shown. In table 3, variables independently associated with hospital mortality according to each component of the PIRO concept are described. Variables retained in the final model for predisposing factors (“P”) included age, gender, previous antibiotic therapy, chronic hepatic disease, chronic hematologic disease, cancer, atherosclerosis and a Karnofsky index <70. For those characterizing infection, (“I”), only the type of infection was retained. Response (“R”) variables included abnormal temperature, tachypnea, hyperglycemia, and the severity of infection. Organ dysfunction (“O”) was characterized by hypotension and a SOFA score ≥1.

**Table 2:** Association of variables of each of the four components of PIRO with hospital mortality using logistic regression. Characteristics of the patients included in the study according to the four components of the PIRO concept.

Predisposition	Total, n (%)	Non-survivors, n (%)		Crude OR	p- value		
Age					<0.001		
≤60 years	388 (38)	18 (5)		1.0			
61–80 years	387 (37)	63 (16)		4.0			
>80 years	260 (25)	57 (22)		5.8			
Male sex, n (%)	506 (49)	79 (16)		1.5	0.036		
Season							
Spring, n (%)	260 (25)	25 (10)		1.0			
Summer, n (%)	248 (24)	32 (13)		1.4	0.242		
Autumn, n (%)	277 (27)	42 (15)		1.7	0.056		
Winter, n (%)	249 (24)	39 (16)		1.7	0.041		
Previous antibiotic therapy, n (%)	367 (36)	67 (18)		1.9	0.001		
Hospitalization in the previous year, n (%)	413 (40)	65 (16)		1.4	0.064		
Previous instrumentation, n (%)	373 (36)	67 (18)		1.8	0.001		
Comorbidities, n (%)	671 (65)	108 (16)		2.1	<0.001		
Immunosupression, n (%)	221 (21)	24 (11)		0.7	0.224		
Chronic hepatic disease, n (%)	22 (2)	8 (36)		3.9	0.003		
Chronic renal disease, n (%)	69 (7)	3 (4)		0.3	0.023		
Chronic heart failure, n (%)	74 (7)	12 (16)		1.3	0.450		
Chronic respiratory disease, n (%)	66 (6)	10 (15)		1.2	0.654		
Chronic haematologic disease, n (%)	60 (6)	17 (28)		2.8	0.001		
Cancer, n (%)	45 (4)	18 (40)		4.8	<0.001		
Diabetes, n (%)	204 (20)	15 (7)		0.5	0.006		
Atherosclerosis, n (%)	242 (23)	54 (22)		2.4	<0.001		
Karnovsky index<70, n (%)	319 (31)	81 (25)		3.9	<0.001		
**Infection**							
Type of infection					0.001		
Community-acquired, n (%)	493 (48)	47 (10)		1.0			
Healthcare-associated, n (%)	225 (22)	32 (14)		1.6			
Hospital-acquired, n (%)	316 (30)	59 (19)		2.2			
Focus of infection					0.140		
Respiratory, n (%)	419 (40)	63 (15)		1.0			
Urinary, n (%)	344 (33)	35 (10)		0.6			
Intra-abdominal, n (%)	213 (21)	29 (14)		0.9			
Other, n (%)	59 (6)	11 (19)		1.3			
Primary bacteraemia, n (%)	57 (6)	10 (17)		1.4	0.338		
Secondary bacteraemia, n (%)	96 (9)	15 (16)		1.3	0.489		
Microbiology isolation, n (%)	703 (68)	99 (14)		1.2	0.303		
Positive blood cultures, n (%)	154 (15)	25 (16)		1.3	0.252		
Type of microorganism, n(%)					0.406		
Gram negative, n (%)	384 (55)	48 (12)		1.0			
Gram positive, n (%)	204 (29)	34 (17)		1.4			
Fungi, n (%)	15 (2)	1 (7)		0.5			
Polymicrobial, n (%)	100 (14)	16 (16)		1.3	0.410		
**Response**							
Temperature						0.006	
No alteration, n(%)	461 (45)	57 (12)		1.0			
Fever, n (%)	336 (33)	35 (10)		0.8			
Hypothermia, n (%)	238 (22)	46 (19)		1.7			
Tachypneia, n (%)	457 (44)	83 (18)		2.1		<0.001	
Tachycardia, n (%)	620 (60)	96 (15)		1.6		0.013	
Reactive C protein>5 mg/dl, n (%)	923 (89)	126 (14)		1.3		0.389	
White blood cells							0.360
No alteration, n (%)	425 (41)	56 (13)	1.0				
Leucocytosis, n (%)	560 (54)	72 (13)	1.0				
Leucopenia, n (%)	50 (5)	10 (20)	1.7				
Altered conscious, n (%)	43 (4)	14 (33)	3.4				<0.001
Hyperglycemia, n (%)	159 (15)	38 (24)	2.4				<0.001
Severity of infection							<0.001
Infection, n (%)	281 (27)	20 (7)	1.0				
Sepsis, n (%)	364 (35)	30 (8)	1.2				
Severe sepsis, n (%)	296 (29)	46 (15)	2.4				
Septic shock, n (%)	94 (9)	42 (45)	10.5				
**Organ dysfunction**							
Hypoxemia, n (%)	267 (26)	57 (21)	2.3				<0.001
Hypotension, n (%)	175 (17)	63 (36)	5.9				<0.001
Lactacidemia>1 mmol/L, n (%)	134 (13)	44 (33)	4.2				<0.001
Creatinine>2 mg/dl or diuresis<0,5 ml/Kg/h, n (%)	136 (13)	32 (23)	2.3				<0.001
Bilirubin>4 mg/dl, n (%)	20 (2)	5 (25)	2.2				0.131
Platelets<100.000, n (%)	96 (9)	26 (27)	2.7				<0.001
Ileus, n (%)	5 (1)	3 (60)	9.9				0.012
Coagulopathy (INR>1.5 or aPTT>60 s), n (%)	8 (1)	4 (3)	6.7				0.008
SOFA>0	691 (67)	118 (17)	3.3				<0.001

SOFA - Sepsis-related Organ Failure Assessment, OR – *Odds ratio.*

**Table 3:** Selection of variables significantly associated with hospital mortality using logistic regression, within each of the four components of PIRO.

Variables	Total, n(%)	Non-survivors, n (%)	Regression coefficients	Adjusted OR	CI_95%_	p- value
**Predisposition**						
Age						
≤60 years	388 (38)	18 (5)		1.0		
61–80 years	387 (37)	63 (16)	0.7	2.0	1.5–2.7	<0.001
>80 years	260 (25)	57 (22)	1.4	4.0	2.2–7.3	<0.001
Male sex, n (%)	506 (49)	79 (16)	0.6	1.8	1.2–2.6	0.005
Previous antibiotic therapy, n (%)	367 (36)	67 (18)	0.7	1.9	1.3–2.9	0.001
Chronic hepatic disease, n (%)	22 (2)	8 (36)	1.9	7.0	2.5–19.0	<0.001
Chronic haematologic disease, n (%)	60 (6)	17 (28)	1.5	4.3	2.2–8.5	<0.001
Cancer, n (%)	45 (4)	18 (40)	1.7	5.6	2.8–11.1	<0.001
Atherosclerosis, n (%)	242 (23)	54 (22)	0.5	1.6	1.0–2.4	0.050
Karnovsky index<70, n (%)	319 (31)	81 (25)	0.9	2.4	1.6–3.8	<0.001
**Infection**						
Community-acquired, n (%)	493 (48)	47 (10)		1.0		
Healthcare-associated, n (%)	225 (22)	32 (14)	0.5	1.6	1.0–2.5	0.064
Hospital-acquired, n (%)	316 (30)	59 (19)	0.8	2.2	1.4–3.3	<0.001
**Response**						
*Temperature*						
No alteration, n(%)	461 (45)	57 (12)		1.0		
Fever, n (%)	336 (33)	35 (10)	−0.4	0.7	0.4–1.1	
Hypothermia, n (%)	238 (22)	46 (19)	0.3	1.4	0.9–2.1	
Tachypneia, n (%)	457 (44)	83 (18)	0.4	1.5	1.0–2.3	0.049
Hyperglycemia, n (%)	159 (15)	38 (24)	0.6	1.7	1.1–2.8	0.016
*Severity of infection*						
Infection or sepsis, n (%)	645 (62)	50 (8)		1.0		
Severe sepsis, n (%)	296 (29)	46 (15)	0.7	1.9	1.2–3.0	0.005
Septic shock, n (%)	94 (9)	42 (45)	2.0	7.4	4.4–12.6	<0.001
**Organ dysfunction**						
Hypotension, n (%)	175 (17)	63 (36)	1.6	4.6	3.1–7.1	<0.001
SOFA>0	691 (67)	118 (17)	0.7	2.0	1.2–3.4	0.009

SOFA – Sepsis-related Organ Failure Assessment, OR – *odds ratio*, CI – confidence interval.

The AU-ROC(CI_95%_) of predicted probabilities for hospital mortality for each PIRO component and the combined PIRO model, in the derivation and in the validation cohorts, are shown in table 4. The combined PIRO model had an AU-ROC of 0.85(0.82–0.88) in the derivation cohort and 0.84 (0.76–0.91) in the validation cohort. The Hosmer and Lemeshow test did not show evidence for lack of fit in all four components or in the combined model, be it in the derivation or in the validation cohorts (p>0.1). Comparing this new PIRO model with SAPS II, it shows a higher discrimination power, with an AU-ROC of 0.85 *vs* 0.81. The performance was also different according to different settings: the PIRO score performed better in the ward with an AU-ROC of 0.84 *vs* 0.78 of the SAPS II while in the ICU setting both had a similar performance, AU-ROC = 0.83 (table 4).

**Table 4:** Area under the receiver operating characteristics curve (95% Confidence Interval) of predicted probabilities by hospital mortality of each PIRO component, the combined PIRO model and SAPS II, in the derivation and in the validation cohorts.

	Predisposition	Insult	Response	Organ	PIRO	SAPS II
**Study population** (n = 1035)	**0.79** (0.75–0.83)	**0.59** (0.54–0.64)	**0.72** (0.67–0.77)	**0.71** (0.66–0.75)	**0.85** (0.82–0.88)	**0.81** (0.77–0.84)
Ward (n = 886)	**0.84** (0.80–0.88)	**0.61** (0.55–0.67)	**0.66** (0.60–0.72)	**0.66** (0.60–0.72)	**0.84** (0.80–0.88)	**0.78** (0.74–0.83)
ICU (n = 149)	**0.77** (0.69–0.86)	**0.50** (0.40–0.60)	**0.79** (0.71–0.88)	**0.70** (0.61–0.79)	**0.83** (0.75–0.91)	**0.83** (0.76–0.91)
**Validation cohort** (n = 186)	**0.75** (0.67–0.83)	**0.60** (0.49–0.70)	**0.73** (0.64–0.83)	**0.77** (0.69–0.85)	**0.84** (0.76–0.91)	**0.85** (0.78–0.92)

**Figure 1:**
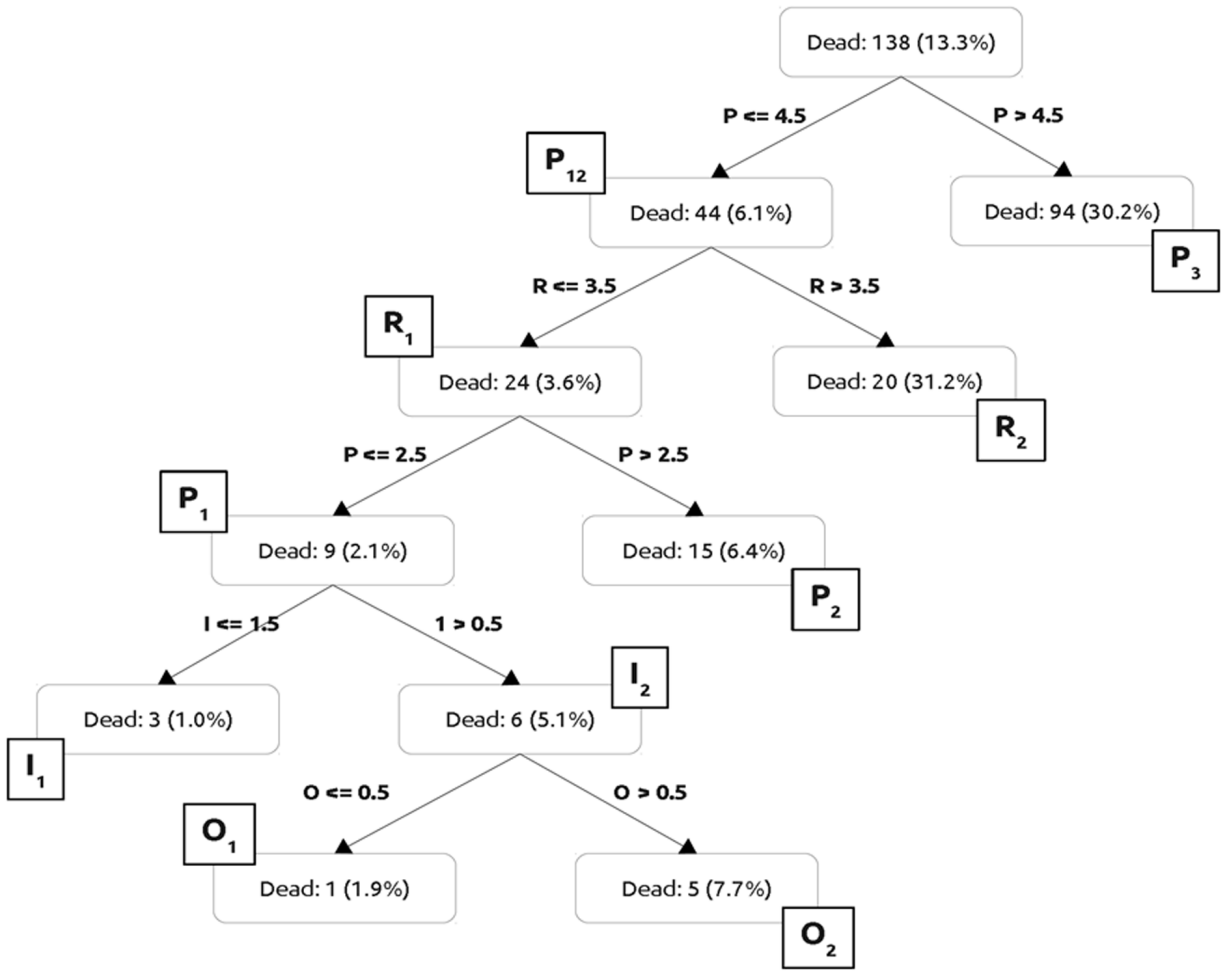
Classification tree used to define cut-offs for component score and identify profiles of risk of death in the derivation cohort across the four PIRO components. Each node split decision in the tree was chosen from the possible cut-offs for all components, maximizing the within-node homogeneity.

Using the rounded regression coefficients for each variable, a weighted clinical classification rule was generated to yield the PIRO scores for each component (table 5). Figure 1 shows the “Classification tree used to define cut-offs for each score and identify clusters of risk of death in the derivation cohort”, allowing patients’ stratification in risk stages for each variable. Predisposition had three stages: P1(0–2 points), P2(3–4 points) and P3(≥5 points). Infection had two stages: I1(0–1 points) and I2(2 points). Response had two stages: R1(0–3 points) and R2(≥4 points). Organ dysfunction had two stages: O1(0 points) and O2(≥1 points).

**Table 5:** Scores attributed to the selected variables regarding each of the four components of PIRO.

P score	Points	I score	Points	R score	Points	O score	Points
*Age*		*Type of infection*		*Altered temperature*		Hypotension	3
≤60 years	0	CAI	0	No	0	SOFA>0	1
61–80	1	HCAI	1	Fever	−1		
>80	3	HAI	2	Hypothermia	1		
Male	1			Hyperglycemia	1		
Previous ATB	1			Tachypneia	1		
Chronic hepatic disease	4			*Severity of infection*			
Chronic haematologic disease	3			Infection or sepsis	0		
Cancer	3			Severe sepsis	1		
Atherosclerosis	1			Septic shock	4		
Karnovsky<70	2						
TOTAL possible points	18		2		7		4

P score – Predisposition score, I score – Insult/Infection score, R score – Host Response score, O score – Organ dysfunction score, ATB – antibiotic therapy, CAI – community-acquired infection, HCAI – healthcare-associated infection, HAI – hospital acquired infection, SOFA – Sepsis-related Organ Failure Assessment.

Increasing stages were associated with an increase in hospital mortality rate, both in the derivation and in the validation cohorts (table 6). The expected mortality was then computed for all possible PIRO combinations defining patients’ risk profiles (Table S1 - Mortality rate and clinical stage according to patients’ PIRO characteristics, in the derivation cohort). Using this table, the profiles were clustered into four stages of increased risk for hospital mortality (Table 7).

**Table 6:** Risk of hospital mortality according to the total score of each PIRO component.

	Predisposition			Insult		Response		Organ	
Risk of mortality classification	P_1_ Low	P_2_ Medium	P_3_ High	I_1_ Low	I_2_ High	R_1_ Low	R_2_ High	O_1_ Low	O_2_ High
*Score (total sum of points)*	*0–2*	*3–4*	*≥5*	*0–1*	*2*	*0–3*	*≥4*	*0*	*≥1*
Derivation Cohort									
Hospital mortality (n = 138)	3%	11%	30%	11%	19%	11%	47%	5%	17%
Percentage of total patients (n = 1035)	45%	25%	30%	69%	31%	93%	7%	33%	67%
Validation Cohort									
Hospital mortality (n = 34)	3%	16%	33%	16%	26%	12%	52%	0%	24%
Percentage of total patients (n = 186)	36%	24%	40%	73%	27%	84%	16%	24%	76%

**Table 7:** Clinical staging system for hospital mortality in patients with infection according to the total score in each PIRO component in the derivation cohort (n = 1035).

Stage I (n = 436)	Stage II (n = 354)	Stage III (n = 197)	Stage IV (n = 42)
Predicted hospital mortalityrate 0%–5%	Predicted hospital mortalityrate 6%–20%	Predicted hospital mortalityrate 21%–50%	Predicted hospital mortalityrate 51%–100%
P_1–2_ I_1–2_ R_1_ O_1_	P_1_ I_2_ R_1_ O_2_	P_3_ I_1–2_ R_1_ O_2_	P_2–3_ I_1–2_ R_2_ O_2_
P_1_ I_1_ R_1_ O_2_	P_1_ I_1_ R_2_ O_2_		
	P_2_ I_1−2_ R_1_ O_2_		
	P_3_ I_1−2_ R_1_ O_1_		
Observed hospital mortality rate = 2% (CI_95% = _0.4–3%)	Observed hospital mortality rate = 11% (CI_95%_ = 8–15%)	Observed hospital mortality rate = 31% (CI_95%_ = 25–37%)	Observed hospital mortality rate = 71% (CI_95% = _58–85%)

Only states with more than 5 patients were included in the different stages. CI – confidence interval.

Stage I (defined as [P_1−2_ I_1−2_ R_1_ O_1_] or [P_1_ I_1_ R_1_ O_2_]) included 436 patients with low or medium predisposition, low response score (without septic shock), and either no organ dysfunction, regardless of place of acquisition, or with organ dysfunction but without hospital-acquired infection. Patients in stage I had a hospital mortality rate of 2% (CI_95%_, 0.4–3%).

Stage II ([P_1_ I_1_ R_2_ O_2_], [P_1_ I_2_ R_1_ O_2_]_,_, [P_2_ I_1−2_ R_1_ O_2_] or [P_3_ I_1−2_ R_1_ O_1_]) included 354 patients with a low predisposition, without hospital-acquired infection, but with a high response score and organ dysfunction or low predisposition, with hospital-acquired infection, low response (without septic shock) but with organ dysfunction. This stage also included patients with medium predisposition score with low response (no septic shock) and organ dysfunction or high predisposition with low response and no organ dysfunction. This group of patients had a hospital mortality rate of 11% (CI_95%_, 8–15%).

The 197 patients in stage III, ([P_3_ I_1–2_ R_1_ O_2_]) were patients with high predisposition, low response and with organ dysfunction. These patients had a hospital mortality rate of 31% (CI_95%_, 25–37%).

Stage IV ([P_2–3_ I_1–2_ R_2_ O_2_]) included 42 patients with a medium or high predisposition score, a high response and also organ dysfunction, regardless of place of acquisition of infection. Their hospital mortality rate was 71% (CI_95%_, 58–85%).

In the validation cohort, the mortality rate was 0% in stage I (0/52), 9% in stage II (5/54), 31% in stage III (15/49) and 67% in stage IV (6/9) (Table S2 - Mortality rate and clinical stage according to patients PIRO characteristics, in the validation cohort).

**Figure 2:**
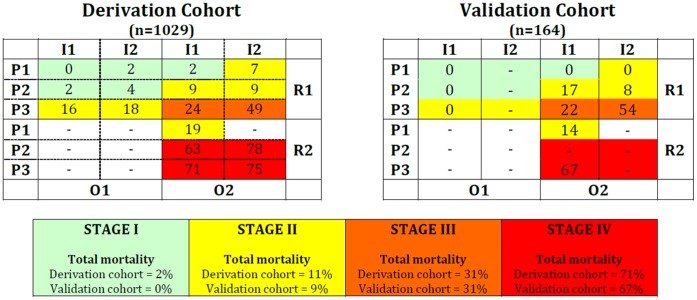
Different stages of PIRO obtained according to the different combinations of Predisposition (P1, P2, P3), Insult (I1, I2), Response (R1, R2) and Organ dysfunction scores (O1, O2). The numbers represent the mortality rate for each state, e.g., the state P_1_I_1_R_2_O_2_ had a mortality of 19% in the derivation cohort and 14% in the derivation cohort. Only states with more than 5 patients were considered. The dashes (−) indicate that there was not enough patients in the respective state to evaluate mortality.

In figure 2 different stages of PIRO obtained according to the different combinations of Predisposition (P1, P2, P3), Insult (I1, I2), Response (R1, R2) and Organ dysfunction scores (O1, O2) are shown.

## Discussion

This study proposes a clinical staging system for patients with infection based on the PIRO concept. It was developed and validated in a large cohort of unselected hospitalized patients with infection because most patients with infection are outside the ICU setting (86% in our study), widening the clinical application of the original concept proposed.

Comparing this new PIRO score with other prognostic scores, namely SAPS II, it performed superiorly (0.85 vs 0.81) with a higher discrimination power, especially in the large sub-group of patients allocated into the ward (0.84 vs 0.78).

Another study [21] has also compared the performance of another PIRO score [11] with APACHE II [14] and MEDS [22] scores in patients admitted into the emergency department with criteria for early goal directed therapy and the severe sepsis resuscitation bundle (that is patients with severe sepsis) and found that for this group of patients, the discrimination power of PIRO and APACHE II (AU-ROC = 0.71) was better than MEDS (AU-ROC = 0.63). The PIRO score proposed by us, presents an even higher discrimination power (AU-ROC = 0.85); however, it should be noted that different populations are included in both studies, but this staging system preformed equally well in more severe populations as it will be discussed later. Four clinical stages of increased risk of hospital mortality were reached, based on “P” characteristics: age, gender, previous antibiotic therapy, chronic hepatic disease, chronic hematologic disease, cancer, atherosclerosis and a Karnofsky index<70; “I”: type of infection; “R”: abnormal temperature, tachypnea, hyperglycemia, and the severity of infection and “O”: hypotension and a SOFA score≥1.

The validation cohort comprised more severe patients, which can perhaps be explained by seasonal variation, as it included patients admitted in the winter, a period coincident to higher occupation rates of hospital beds with more unscheduled admissions. The higher severity of the patients included in the validation cohort is probably the explanation for the higher mortality rate observed. Nonetheless, even in this cohort of more severe patients, the clinical staging system performed equally well, which is a good indication of the generalizability of the model.

Many interventions tested in clinical trials of critically ill patients with severe sepsis have failed to show benefit. One of the potential reasons for this was an inadequate, non-specific selection of patients enrolled in those trials relying mostly on the original systemic inflammatory response syndrome (SIRS) criteria, with equal weight being given to all variables. A clinical staging system derived from a prognostic model, attributing different weights to each co-variable of the four PIRO components, is more likely to better stratify patients and refine inclusion criteria in such trials.

Previous studies have been limited to selected populations or hospital settings [5], [7]–[11]. Lisboa et al [7] developed a score derived from the PIRO concept to predict ICU mortality in patients with ventilator-associated pneumonia. Rello et al [8], performed a similar study focused only on community acquired pneumonia requiring ICU admission. The present study enriches previous findings by including the three most frequent focus of infection which leads to patients requiring hospital care.

Howell et al [10] developed a scoring system on patients admitted to the emergency department with suspected infection. However, they did not follow subsequent information from the hospital course, so patients ultimately found to have a non-infectious diagnosis may have been included. The only inclusion criteria in the present study was the presence of clinical infection, assessed by the CDC definitions [12] which can be done immediately at bed side, thus not delaying patient stratification and its adequate application in the current study was reinforced by the high microbiological documentation rate, minimizing selection bias.

Moreno et al [9] developed a score in ICU patients, from a subset of patients from the SAPS III database, using a modified definition of PIRO (PIR). They excluded patients who died during the first 48 h, which might exclude patients with high response and organ dysfunction scores (like septic shock), thus underweighting these components in their model. Besides including all patients regardless of the severity of infection, the present study evolves further into a clinical staging system.

Rubulotta et al [11] also performed a secondary analysis of two cohorts of ICU patients (PROWESS and PROGRESS databases) and defined a basic phenotypic characterization of patients admitted with severe sepsis. However, in their retrospective study it was not possible to analyze all variables originally proposed [2]. Being prospective and following a rigorous methodology, including a large cohort of unselected hospitalized patients with infection, the present study has reached a pilot clinical staging system that might include all clinical relevant variables.

Classification trees were used to optimize the discrimination ability of the model rather than determine cut-offs heuristically after a logistic regression. Finally an independent validation cohort was used to assess the robustness of the data and over fitting of the derived model.

Although proponents of the PIRO staging system suggested including biomarkers and/or variables reflecting genetic predisposition, these tools are not yet widely or routinely available. Thus, analyses were derived from covariates currently available at bedside, which might help immediate patient management. Data on microbiology documentation and antibiotics appropriateness were also not included, although they could represent major prognostic factors, they are not readily available for early stratification, which is the main goal of the proposed staging system.

The differences in mortality rates between the cohorts can be explained by random variability due to the small sample size of the validation cohort. However, this is a single-center study with a limited number of patients, both in the derivation and in the validation cohorts; nonetheless, it might represent a major step forward in the clinical application of the PIRO concept, expanding its applicability to all hospitalized patients with infection. Further validation in different settings is needed.

In conclusion, it enriches the findings of previous studies by reaching a clinical staging system through its prospective designed with consideration of all proposed variables [2], including patients at various levels of care inside the hospital, with different focus of infection and severity of disease, widening its application to the vast majority of infected patients, with a robust behavior both in the derivation (AU-ROC = 0.85) and in the validation cohort (AU-ROC = 0.84).

At this point, we propose its use mainly after further validation, for early stratification and inclusion in clinical trials. We hope that in the very near future, it can also be useful to tailor individual therapy.

### Conclusions

This study proposes a clinical staging system according to the PIRO concept, with stratification of patients according to their risk of death, derived from different scores obtained in Predisposition considering: age, sex, previous antibiotic therapy, chronic hepatic disease, chronic hematologic disease, cancer, atherosclerosis and a Karnofsky index<70; type of infection in Insult/Infection; abnormal temperature, tachypnea, hyperglycemia and severity of infection in Response and hypotension and SOFA score≥1 in Organ dysfunction. It allowed the building of four stages with increased risk of mortality: from stage I [i.e., P_1_I_1_R_1_O_1_] associated with a low (≤5%) risk of death, to stage IV [i.e., P_3_I_2_R_2_O_2_], where the risk of mortality is highest (>50%).

Staging infected patients according to the four components of the PIRO system may be a practical and relevant tool in sepsis research. In particular, this new clinical staging system for hospital mortality in patients with infection may prove to become a useful triage tool to design individualized management strategies as well as for refining inclusion criteria in clinical trials.

## Supporting Information

Table S1 **Mortality rate and clinical stage according to patients PIRO characteristics, in the derivation cohort. Only states with more than 5 patients were included in the table.**
(DOC)

Table S2 **Mortality rate and clinical stage according to patients PIRO states, in the validation cohort. Only states with more than 5 patients were included in the table.**
(DOC)

## References

[1] WHO (2012) World Health Statistics 2012. In: WHO G, editor. Available: http://apps.who.int/iris/bitstream/10665/44844/1/9789241564441_eng.pdf, Accessed 2010 Jul 1.

[2] Levy MM, Fink MP, Marshall JC, Abraham E, Angus D, et al (2003) 2001 SCCM/ESICM/ACCP/ATS/SIS International Sepsis Definitions Conference. Crit Care Med 31: 1250–1256. 12682500 10.1097/01.CCM.0000050454.01978.3B

[3] Denoix P (1946) Enquete permanent dans les centres anticancerreaux. Bull Inst Natl Hyg 1: 70–75. 20986738

[4] Angus DC, Burgner D, Wunderink R, Mira JP, Gerlach H, et al (2003) The PIRO concept: P is for predisposition. Crit Care 7: 248–251. 12793879 10.1186/cc2193 PMC270687

[5] Adrie C, Francais A, Alvarez-Gonzalez A, Mounier R, Azoulay E, et al (2009) Model for predicting short-term mortality of severe sepsis. Crit Care 13: R72. 19454002 10.1186/cc7881 PMC2717433

[6] Granja C, Povoa P, Lobo C, Teixeira-Pinto A, Carneiro A, et al (2013) The predisposition, infection, response and organ failure (Piro) sepsis classification system: results of hospital mortality using a novel concept and methodological approach. PLoS One 8: e53885. 23349756 10.1371/journal.pone.0053885 PMC3548822

[7] Lisboa T, Diaz E, Sa-Borges M, Socias A, Sole-Violan J, et al (2008) The ventilator-associated pneumonia PIRO score: a tool for predicting ICU mortality and health-care resources use in ventilator-associated pneumonia. Chest 134: 1208–1216. 18779186 10.1378/chest.08-1106

[8] Rello J, Rodriguez A, Lisboa T, Gallego M, Lujan M, et al (2009) PIRO score for community-acquired pneumonia: a new prediction rule for assessment of severity in intensive care unit patients with community-acquired pneumonia. Crit Care Med 37: 456–462. 19114916 10.1097/CCM.0b013e318194b021

[9] Moreno RP, Metnitz B, Adler L, Hoechtl A, Bauer P, et al (2008) Sepsis mortality prediction based on predisposition, infection and response. Intensive Care Med 34: 496–504. 18060541 10.1007/s00134-007-0943-1

[10] Howell MD, Talmor D, Schuetz P, Hunziker S, Jones AE, et al (2011) Proof of principle: the predisposition, infection, response, organ failure sepsis staging system. Crit Care Med 39: 322–327. 21099424 10.1097/CCM.0b013e3182037a8e

[11] Rubulotta F, Marshall JC, Ramsay G, Nelson D, Levy M, et al (2009) Predisposition, insult/infection, response, and organ dysfunction: A new model for staging severe sepsis. Crit Care Med 37: 1329–1335. 19242329 10.1097/CCM.0b013e31819d5db1

[12] Garner JS, Jarvis WR, Emori TG, Horan TC, Hughes JM (1988) CDC definitions for nosocomial infections Am J Infect Control. 16: 128–140. 10.1016/0196-6553(88)90053-3 2841893

[13] Karnofsky DA, Burchenal JH (1949) “The Clinical Evaluation of Chemotherapeutic Agents in Cancer.” In: CM M, editor. Evaluation of Chemotherapeutic Agents Columbia Univ Press. 196.

[14] Knaus WA, Draper EA, Wagner DP, Zimmerman JE (1985) APACHE II: a severity of disease classification system. Crit Care Med 13: 818–829. 3928249

[15] Le GallJR, Lemeshow S, Saulnier F (1993) A new Simplified Acute Physiology Score (SAPS II) based on a European/North American multicenter study. JAMA 270: 2957–2963. 8254858 10.1001/jama.270.24.2957

[16] Friedman ND, Kaye KS, Stout JE, McGarry SA, Trivette SL, et al (2002) Health care–associated bloodstream infections in adults: a reason to change the accepted definition of community-acquired infections. Ann Intern Med 137: 791–797. 12435215 10.7326/0003-4819-137-10-200211190-00007

[17] Horan TC, Emori TG (1997) Definitions of key terms used in NNIS System. Am J Infect Control 25: 112–116. 9113287 10.1016/s0196-6553(97)90037-7

[18] Vincent JL, Moreno R, Takala J, Willatts S, De Mendonca A, et al (1996) The SOFA (Sepsis-related Organ Failure Assessment) score to describe organ dysfunction/failure. On behalf of the Working Group on Sepsis-Related Problems of the European Society of Intensive Care Medicine. Intensive Care Med 22: 707–710. 8844239 10.1007/BF01709751

[19] Gini C, editor (1912) Variabilità e mutabilità (Variability and Mutability). Reprinted in Memorie di metodologica statistica (Ed. Pizetti E, Salvemini, T). Rome: Libreria Eredi Virgilio Veschi (1955) ed. Bologna. 156 p.

[20] Hand DJ, Till RJ (2001) A Simple Generalisation of the Area Under the ROC Curve for Multiple Class Classification Problems. Machine Learning 45: 171–186.

[21] Nguyen HB, Van Ginkel C, Batech M, Banta J, Corbett SW (2011) Comparison of Predisposition, Insult/Infection, Response, and Organ dysfunction, Acute Physiology And Chronic Health Evaluation II, and Mortality in Emergency Department Sepsis in patients meeting criteria for early goal-directed therapy and the severe sepsis resuscitation bundle. J Crit Care 27 (4): 362–9. 10.1016/j.jcrc.2011.08.013 22033054

[22] Shapiro NI, Wolfe RE, Moore RB, Smith E, Burdick E, et al (2003) Mortality in Emergency Department Sepsis (MEDS) score: a prospectively derived and validated clinical prediction rule. Crit Care Med 31: 670–675. 12626967 10.1097/01.CCM.0000054867.01688.D1

