# Supplementary material for: Predisposition, Insult/Infection, Response and Organ Dysfunction (PIRO): A Pilot Clinical Staging System for Hospital Mortality in Patients with Infection
Source: PLoS One. 2013 Jul 24;8(7):e70806. doi: 10.1371/journal.pone.0070806 (PMC3722163; doi:10.1371/journal.pone.0070806)
Supplement: Table S1 — Mortality rate and clinical stage according to patients PIRO characteristics, in the derivation cohort. Only states with more than 5 patients were included in the table. (DOC) [file pone.0070806.s001.doc]

**Table S1–Mortality rate and clinical stage according to patients PIRO characteristics, in the derivation cohort. Only states with more than 5 patients were included in the table.**

|  |  | Mortality | | Total |
| --- | --- | --- | --- | --- |
| PIRO STATES | Clinical stage | n | (%) | n |
| P1 I1 R1 O1 | I | 0 | (0) | 129 |
| P1 I1 R1 O2 | I | 4 | (2) | 185 |
| P1 I2 R1 O1 | I | 1 | (2) | 52 |
| P2 I1 R1 O1 | I | 1 | (2) | 44 |
| P2 I2 R1 O1 | I | 1 | (4) | 26 |
| P1 I1 R2 O2 | II | 5 | (19) | 26 |
| P1 I2 R1 O2 | II | 5 | (7) | 69 |
| P2 I1 R1 O2 | II | 10 | (9) | 113 |
| P2 I2 R1 O2 | II | 5 | (9) | 57 |
| P3 I1 R1 O1 | II | 9 | (16) | 56 |
| P3 I2 R1 O1 | II | 6 | (18) | 33 |
| P3 I1 R1 O2 | III | 33 | (24) | 140 |
| P3 I2 R1 O2 | III | 28 | (49) | 57 |
| P2 I1 R2 O2 | IV | 5 | (63) | 8 |
| P2 I2 R2 O2 | IV | 7 | (78) | 9 |
| P3 I1 R2 O2 | IV | 12 | (71) | 17 |
| P3 I2 R2 O2 | IV | 6 | (75) | 8 |
|  |  |  |  |  |
|  | Total I | 7 | (2) | 436 |
|  | Total II | 40 | (11) | 354 |
|  | Total III | 61 | (31) | 197 |
|  | Total IV | 30 | (71) | 42 |
|  | TOTAL | 138 | (13) | 1029 |
