# Supplementary material for: Predisposition, Insult/Infection, Response and Organ Dysfunction (PIRO): A Pilot Clinical Staging System for Hospital Mortality in Patients with Infection
Source: PLoS One. 2013 Jul 24;8(7):e70806. doi: 10.1371/journal.pone.0070806 (PMC3722163; doi:10.1371/journal.pone.0070806)
Supplement: Table S2 — Mortality rate and clinical stage according to patients PIRO states, in the validation cohort. Only states with more than 5 patients were included in the table. (DOC) [file pone.0070806.s002.doc]

**Table S2–Mortality rate and clinical stage according to patients PIRO states, in the validation cohort. Only states with more than 5 patients were included in the table.**

|  |  | Mortality | | Total |
| --- | --- | --- | --- | --- |
| PIRO STATES | Clinical stage | n | (%) | n |
| P1I1R1O1 | I | 0 | (0) | 20 |
| P1I1R1O2 | I | 0 | (0) | 25 |
| P2I1R1O1 | I | 0 | (0) | 7 |
| P1I1R2O2 | II | 1 | (14) | 7 |
| P1I2R1O2 | II | 0 | (0) | 8 |
| P2I1R1O2 | II | 3 | (17) | 18 |
| P2I2R1O2 | II | 1 | (8) | 13 |
| P3I1R1O1 | II | 0 | (0) | 8 |
| P3I1R1O2 | III | 8 | (22) | 36 |
| P3I2R1O2 | III | 7 | (54) | 13 |
| P3I1R2O2 | IV | 6 | (67) | 9 |
|  |  |  |  |  |
|  |  |  |  |  |
|  |  |  |  |  |
|  | Total I | 0 | (0) | 52 |
|  | Total II | 5 | (9) | 54 |
|  | Total III | 15 | (31) | 49 |
|  | Total IV | 6 | (67) | 9 |
|  | Total | 26 | (16) | 164 |
|  |  |  |  |  |
|  |  |  |  |  |
|  |  |  |  |  |
|  |  |  |  |  |
